# 국내 거주 베트남 여성의 인유두종바이러스 백신접종 의도의 영향요인: 횡단적 상관관계 연구

**DOI:** 10.7586/jkbns.25.065

**Published:** 2025-11-26

**Authors:** Haewon Lee, Seungmi Park

**Affiliations:** 1 National Health Insurance Service, Cheongju, Korea; 1 국민건강보험공단; 2 Department of Nursing Science·Research Institute of Nursing Science, College of Nursing, Chungbuk National University, Cheongju, Korea; 2 충북대학교 간호대학·간호과학연구소

**Keywords:** Human papilloma virus, Papillomavirus vaccines, Health belief model, Emigrants and immigrants, Vietnam, 사람유두종 바이러스, 유두종 바이러스 백신, 건강신념모델, 이민자와 이주민, 베트남

## Abstract

**Purpose:**

This study aimed to assess the level of health beliefs among Vietnamese women residing in Korea using the health belief model and to identify factors influencing their intention to receive the human papillomavirus (HPV) vaccine. The ultimate goal was to suggest potential intervention strategies to improve vaccination uptake.

**Methods:**

This descriptive survey was conducted with 146 Vietnamese women residing in the Chungbuk area using a self-reported questionnaire. Data were collected from January 16 to December 26, 2024. Statistical analyses were performed using SPSS version 27.0, employing descriptive statistics, the independent t-test, one-way analysis of variance, Pearson correlation coefficients, and multiple regression analysis.

**Results:**

Self-efficacy, residential period in Korea, perceived barriers, and perceived susceptibility were identified as factors influencing HPV vaccination intention among Vietnamese migrant women.

**Conclusion:**

The results of this study suggest the need for comprehensive interventions, including customized education programs and institutional support, to improve HPV vaccination rates among Vietnamese women residing in South Korea.

## 서론

### 1. 연구의 필요성

자궁경부암은 자궁의 입구인 자궁경부에 발생하는 여성 생식기 암으로 자궁경부암의 위험요인은 생식기 감염을 일으키는 가장 흔한 바이러스인 인유두종바이러스(human papilloma virus, HPV)를 비롯하여 생활요인과 환경요인 및 유전요인 등 여러 요인이 복합적으로 관여하여 발생하는 암으로 전 세계 여성에게 네 번째로 많은 암이자, 우리나라 여성의 암유병자분율에 갑상선, 유방, 대장, 위암 이후로 5위의 4.7% 차지하고 있다[1]. 2006년에 세계 최초로 HPV 백신이 승인되었으며, 우리나라는 2016년 자궁경부암 예방접종을 도입하여 만 12세~17세의 여성청소년에게 접종을 시작하여 현재 가다실 4가, 9가와 서바릭스 백신이 국내 승인되어 있다[2,3]. 그러나 많은 국가들이 아직 HPV 백신을 국가 예방접종으로 도입하지 않고 있다. 특히, HPV 백신을 국가 예방접종 프로그램으로 시행하고 있지 않은 나라인 베트남은 2019년부터 지속적으로 국내 체류 외국인 수에서 2위를 차지하고 있는 국가로 2022년에는 235,007명의 베트남인이 체류 중이며, 2022년 국내의 국제결혼 통계에서 베트남인과의 국제결혼 부부가 가장 많은 것으로 발표되었다[4,5]

자궁경부암은 2018년 베트남 여성 암 중 6번째로 많은 질환이다. 베트남에서는 2020년 기준, 자궁경부암의 위험이 있는 15세 이상 여성 인구가 3,910만 명이며 매년 4,132명의 여성이 자궁경부암 진단을 받고, 2,223명이 사망하였지만, 여전히 베트남은 아직 HPV 백신을 국가 예방접종을 시행하지 않고 있는 나라 중 한 곳이다[6]. 2021년 베트남의 15~29세의 여성 중 12%, 30~49세 여성 중 28%, 소수의 여성만이 HPV 예방접종을 받았다[7]. 베트남의 여대생을 대상으로 한 연구에서 절반 이상이 HPV와 예방접종에 대해 들어본 적이 있었지만, 실제로 예방접종을 받은 사람은 5% 미만이었다[8]. 또한, 베트남은 아직 자궁경부암 정기적인 스크리닝 검사 제도가 도입되지 않아 위험 수준이 충분히 파악되지 않고 있다.

이러한 상황에서 개인의 건강행동을 유발하는 것에 대한 이론적 모형인 건강신념모형(Health Belief Model)이 활용될 수 있다. 건강신념모형은 지각된 민감성, 지각된 심각성, 지각된 유익성, 지각된 장애성, 행동 계기, 자기효능감으로 구성되어 있다[9]. 건강신념모형을 활용한 연구는 건강신념의 하부개념에 따른 건강 행위의 변화를 끌어낼 수 있어 건강신념이 HPV 백신 접종 의도 미치는 영향요인을 확인할 필요가 있다.

베트남 여성을 대상으로 한 선행연구의 대상자는 대부분 결혼이주여성에 국한되어있었다. 선행연구에 따르면 결혼이주여성은 의료기관을 이용할 때 배우자 의존도가 높은 편이며, 이주여성들이 한국에 거주하며 생활하면서 가장 의지해야 할 가족이 배우자임에도 이주여성 배우자의 자궁경부암 및 HPV 백신에 대한 지식수준은 국내의 남성 대학생과 비교하여 높지 않은 편으로 확인되었다[10,11]. 행동의 직접적인 결정요인이 되는 의도를 파악하기 위하여[12], 결혼이주여성 대상으로 한 HPV 백신 지식, 태도와 예방접종 의도의 영향요인을 파악하거나, 자궁경부암 지식과 HPV 예방접종 건강신념 정도를 확인한 연구와 결혼이주여성 자녀의 HPV 예방접종 영향요인을 조사한 연구가 수행되어왔다[13-15].

국내에서 이루어진 베트남 여성 관련 선행연구는 대부분 결혼이주여성에 초점을 맞추고 있어, 국내 거주 베트남 여성 전반의 건강요인을 충분히 설명하기에는 한계가 있다. 그러나 한월혼혈 인구가 꾸준히 증가하고 있으며[5], 이에 따라 단순한 체류 외국인 집단이 아닌 국내 사회의 구성원으로서의 베트남 여성 집단이 공중보건학적으로 갖는 중요성은 점차 커지고 있다. 그럼에도 불구하고 베트남 여성 전반을 대상으로 한 HPV 예방접종 관련 연구는 거의 이루어지지 않았기에 HPV 예방접종에 대한 신념과 접종 의도 관계성을 파악하는 연구는 의의가 있다. 베트남 여성들은 자국에서도, 우리나라에서도 HPV 예방접종의 대상에도 해당하지 않아 자궁경부암 등 HPV 감염의 위험성은 높은 실정이다. 이에 건강신념모형에 근거하여 베트남 여성의 HPV 예방접종 의도에 영향을 미치는 요인을 분석함으로써 확대가 불가피한 다문화 사회에서 베트남 여성의 건강증진을 위한 기초자료를 마련하고자 한다.

### 2. 연구의 목적

1) 연구대상자의 일반적 특성 및 HPV, 성경험 관련 특성을 파악한다.

2) 연구대상자의 HPV 예방접종에 대한 건강신념과 예방접종 의도 정도를 파악한다.

3) 연구대상자의 특성에 따른 HPV 예방접종에 대한 건강신념 정도와 예방접종 의도의 차이를 파악한다.

4) 연구대상자의 HPV 예방접종 관련 신념 요인과 예방접종 의도 간의 상관관계를 파악한다.

5) 연구대상자의 HPV 예방접종 의도에 영향을 미치는 요인을 확인한다.

## 연구 방법

### 1. 연구 설계

본 연구는 국내 거주 베트남 여성의 HPV 예방접종 관련 건강신념이 예방접종 의도에 미치는 영향을 파악하기 위한 서술적 조사연구이다.

### 2. 연구 대상

본 연구의 대상자는 국내에 실거주지를 두고 거주하고 있는 본 국적이 베트남인 여성을 대상으로 수행하였으며, 연구의 목적을 이해하고 자발적으로 연구 참여에 동의한 자이다. 연구대상자 수는 G*power 3.1.9.7 프로그램[16]을 이용하여 산출한 결과, HPV 예방접종에 대한 선행연구[10,17-21]에서 유리한 변인으로 나타났던 건강신념 하위영역 5개의 항목(지각된 민감성, 지각된 심각성, 지각된 유익성, 지각된 장애성, 자기효능감)과 더미 변수의 수를 고려하여 예측요인 11개를 고려한 최소 표본 수는 135명이었으며, 중도 탈락률 약 20%를 반영하여 총 169명으로 산출되었다. 총 148명이 연구 참여에 동의하여 설문을 완료하였고, 중복응답 등의 사유로 2명을 제외한 146명의 자료를 자료 분석에 활용하였다.

### 3. 연구 도구

#### 1) 대상자의 일반적 특성

본 연구에서 대상자의 일반적 특성은 연령, 한국 거주기간, 최종학력, 결혼상태, 자녀 여부, 경제적 수준, 성 특성 등으로 구성하였다.

#### 2) 대상자의 지각된 민감성

지각된 민감성은 개인이 질병에 관해 예민하게 느끼거나 질병에 걸릴 수 있다고 느끼는 주관적인 위험의 정도로 곧 질환에 걸릴 가능성에 대해 개인이 지각하는 정도를 의미하며[22], Kim과 Cho [23]가 개발한 도구를 Zhang [24]이 HPV 예방접종의 건강신념 측정을 위해 수정한 도구를 사용하였다. 도구는 총 5문항으로 각 문항은 1점 ‘매우 그렇지 않다(1점)’부터 ‘매우 그렇다(5점)’의 5점 Likert 척도로 점수가 높을수록 민감성이 높음을 의미한다. 도구의 신뢰도는 Zhang [24]의 연구에서 .92이었으며, 본 연구에서는 .81이었다.

#### 3) 대상자의 지각된 심각성

지각된 심각성은 질병에 걸렸을 때 신체적, 사회적 측면에서 나타나는 결과의 심각성에 대해 개인이 지각하는 정도를 의미하며[22], Kim과 Cho [23]가 개발한 도구를 Zhang [24]이 HPV 예방접종의 건강신념 측정을 위해 수정한 도구를 사용하였다. 도구는 총 4문항으로 각 문항은 1점 ‘매우 그렇지 않다(1점)’부터 ‘매우 그렇다(5점)’의 5점 Likert 척도로 점수가 높을수록 심각성이 높음을 의미한다. 도구의 신뢰도는 Zhang [24]의 연구에서 .94이었으며, 본 연구에서는 .79이었다.

#### 4) 대상자의 지각된 유익성

지각된 유익성은 질환의 위협이나 민감성을 감소시키는 데 효과적이라고 지각하는 정도를 말하며[22], Hong 등[25]의 연구에서 개발된 도구를 Zhang [24]이 HPV 예방접종의 건강신념 측정을 위해 수정한 도구를 사용하였다. 도구는 총 5문항으로 각 문항은 1점 ‘매우 그렇지 않다(1점)’부터 ‘매우 그렇다(5점)’의 5점 Likert 척도로 점수가 높을수록 유익성이 높음을 의미한다. 도구의 신뢰도는 Zhang [24]의 연구에서 .94이었으며, 본 연구에서는 .77이었다.

#### 5) 대상자의 지각된 장애성

지각된 장애성은 건강 관련 예방행위를 실천하는데 있어 방해하는 심리적, 상황적 요인들에 대한 불편함을 지각하는 정도를 의미하며[22], Hong 등[25]의 연구에서 개발된 도구를 Zhang [24]이 HPV 예방접종의 건강신념 측정을 위해 수정한 도구를 사용하였다. 도구는 총 6문항으로 1점 ‘매우 그렇지 않다(1점)’부터 ‘매우 그렇다(5점)’의 5점 Likert 척도로 점수가 높을수록 장애성이 높음을 의미한다. 도구의 신뢰도는 Zhang [24]의 연구에서 .95이었으며, 본 연구에서는 .74이었다.

#### 6) 대상자의 자기효능감

자기효능감은 특정 과업이나 목적을 달성할 능력에 대한 기대이자, 개인의 사고과정, 스스로의 동기, 감정상태, 행동유형 등을 통제할 수 있다고 믿는 신념의 정도를 의미하며[26], Cho [27]와 Jo 등[28]이 개발한 도구를 Nguyen과 Lee [29]가 HPV 예방접종의 건강신념 측정을 위해 수정한 도구 사용하였다. 도구는 총 3문항으로 ‘매우 그렇지 않다(1점)’부터 ‘매우 그렇다(5점)’의 5점 Likert 척도로 점수가 높을수록 자기효능감이 높음을 의미한다. 도구의 신뢰도는 Nguyen과 Lee [29]의 연구에서 .91이었으며, 본 연구에서는 .80이었다.

#### 7) 대상자의 HPV 예방접종 의도

예방접종 의도는 개인이 특정한 행동을 수행하고자 하는 정도를 의미하며[12], 본 연구에서는 대상자가 HPV 예방접종을 위해 노력하려 하는지, 자발적으로 열심히 하려는 지에 관련된 정도를 의미하는 것으로 ‘나는 HPV 백신 예방접종을 맞을 의향이 있다.’는 한가지 문항으로 구성하였다. ‘전혀 그렇지 않다(1점)’부터 ‘매우 그렇다(5점)’ Likert 5점 척도로 측정하여 점수가 높을수록 예방접종의 의도가 높은 것을 의미한다.

### 4. 자료 수집

자료 수집은 2024년 1월 16일부터 12월 26일까지 진행되었으며, 충북지역의 다문화가족지원센터에 협조를 요청하여 해당 기관의 담당자가 제공하는 설문지 URL을 통해 접속한 자가 자발적으로 연구 참여에 동의한 경우에만 온라인에서 자가 보고식 설문을 입력하는 것으로 자료를 수집하였다. 설문지는 통번역 전문가 1인과 양국의 이중 언어 가능자 1인의 번역과 동등성 비교를 수행한 후, 베트남어로 제공되었다. 항목은 43개로 응답에는 약 15~20분이 소요되었으며, 연구 참여에 도움이 필요한 대상자는 담당자의 지원을 받을 수 있도록 안내하였다.

### 5. 자료 분석

수집된 자료는 SPSS version 27.0 (IBM Corp., Armonk, NY, USA) 통계 프로그램을 이용하여 분석하였다. 연구대상자의 일반적 특성 및 HPV, 성경험 관련 특성과 HPV 예방접종에 대한 건강신념 정도와 예방접종 의도는 빈도와 백분율, 평균과 표준편차를 이용하여 기술 통계로 산출하였다. 연구대상자의 일반적 특성 및 HPV, 성경험 관련 특성에 따른 HPV 예방접종에 대한 건강신념, 예방접종 의도의 차이는 independent t-test와 one-way analysis of variance로 분석하고, 사후검정은 Scheffé test로 확인하였다. HPV 예방접종 관련 건강신념 요인과 예방접종 의도 간의 상관관계는 Pearson correlation coefficient로 분석하였으며, HPV 예방접종 의도에 영향을 미치는 요인은 입력(enter) 방법을 이용하여 다중 회귀분석(multiple regression analysis)을 실시하였다.

### 6. 윤리적 고려

본 연구는 충북대학교 생명윤리심의위원회(Institutional Review Board, IRB)의 승인을 받은 후 연구를 수행하였다(IRB No. CBNU-202312-HR-0251). 대상자에게 연구 참여 전 연구의 필요성과 목적, 기대효과, 자료수집 방법 및 소요시간, 비밀보장, 연구자의 연락처 등의 내용이 포함되었으며, 이에 자발적으로 참여에 동의한 대상자만 온라인에서 설문에 참여할 수 있도록 하였다. 수집된 설문 자료는 모두 전산 입력 작업을 진행하여 기본적인 정보의 안전과 보호를 위해서 설문 조사 참여 순서에 따른 번호를 ID로 부여하였으며, 연구자 본인 이외의 사람이 접근할 수 없도록 비밀번호를 설정하여 보관하였다. 연구논문이 인쇄자료로 발표된 후에는 설문 자료 모두를 삭제하여 폐기할 예정이다.

## 연구 결과

### 1. 대상자의 일반적 특성

대상자의 평균 연령은 32.2 ± 6.08세였으며 30세 미만이 53명(36.3%)과 35세 이상이 53명(36.3%)으로 나타났다. 최종학력은 고졸 이하 66명(45.2%)으로 가장 많았고, 출신 지역은 중앙직할시인 하노이, 호찌민, 하이퐁, 다낭, 껀터 출신이 75명(51.4%)으로 많았다. 결혼 여부는 기혼이 106명(72.6%), 자녀가 있는 대상자가 104명(71.2%)으로 대부분을 차지했다. 한국에서의 평균 거주기간은 8.13 ± 5.02년으로 나타났고, 5년 이상 10년 미만이 61명(41.8%)으로 가장 높은 비율을 보였다. HPV 관련 특성으로 HPV를 알고 있다고 응답한 군이 109명(74.7%), HPV 백신 접종경험이 있는 대상자는 111명(76.0%)이었다. 자궁경부암을 알고 있다고 대답한 군이 133명(91.1%)으로 대부분을 차지하였으며, 자궁경부암 검진 경험이 있다고 응답한 군은 83명(56.8%)으로 나타났다. 성경험 관련 특성으로 성경험이 있다고 응답한 군이 135명(92.5%)으로 대부분이었으며, 첫 성경험 평균 연령은 22.38 ± 2.88세, 성관계 남성 수에 대한 응답으로는 1명이 82명(61.2%)으로 대부분이었다(Table 1).

### 2. HPV 예방접종에 대한 건강신념과 예방접종 의도

대상자의 HPV 예방접종에 대한 건강신념과 예방접종 의도를 분석한 결과, 건강신념의 하위영역 중에서는 자기효능감이 평균 4.29 ± 0.74점으로 가장 높게 나타났고, 지각된 심각성 4.14 ± 0.84점, 지각된 유익성 4.06 ± 0.77점, 지각된 민감성 3.82 ± 0.90점, 지각된 장애성 2.90 ± 0.80점 순으로 나타났으며, 예방접종 의도는 4.52 ± 0.75점이었다(Table 2).

### 3. 일반적 특성 및 HPV, 성경험 관련 특성에 따른 건강신념의 하위영역 별 차이

대상자의 일반적 특성 및 HPV, 성경험 관련 특성에 따른 건강신념의 하위영역 별 차이를 분석한 결과, 지각된 민감성은 HPV 백신 접종 경험(t = 3.02, *p* = .003), 자궁경부암 인지(t = 2.40, *p* = .018), 자궁경부암 검진 경험(t = 2.71, *p* = .008)에 따른 통계적으로 유의한 차이가 있었다. 지각된 심각성과 지각된 유익성은 자궁경부암 검진 경험(t = 3.12, *p* = .002, t = 2.34, *p* = .021)에서 통계적으로 유의한 차이가 있는 것으로 나타났으며, 자기효능감은 첫 성경험 연령에서 통계적으로 유의한 차이가 있었다(F = 3.31, *p* = .040). 지각된 장애성은 일반적 특성 및 HPV, 성경험 관련 특성에 따라 통계적으로 유의한 차이가 없었다(Table 3).

### 4. 일반적 특성 및 HPV, 성경험 관련 특성에 따른 HPV 예방접종 의도의 차이

일반적 특성 및 HPV, 성경험 특성 관련에 따른 HPV 예방접종 의도의 차이를 분석한 결과, 일반적 특성 중 거주기간(F = 3.67, *p* = .028)과 성경험 특성 중 첫 성경험 연령(F = 3.65, *p* = .029)이 통계적으로 유의한 차이가 있는 것으로 나타났다. 사후분석 결과, 거주기간이 5년 미만인 경우보다 5년 이상, 10년 미만인 경우와 첫 성경험 연령이 20세 미만인 경우보다 25세 이상인 경우에 통계적으로 유의하게 높았다. 그러나 HPV 특성에 따라서는 HPV 예방접종 의도에 유의한 차이가 없었다(Table 1).

### 5. HPV 예방접종 관련 건강신념 하부요인과 예방접종 의도의 상관관계

HPV 예방접종 관련 건강신념 하부요인과 예방접종 의도의 상관관계를 분석한 결과, HPV 백신의 예방접종 의도는 건강신념의 하부요인 중 지각된 민감성(r = .35, *p* < .001), 지각된 심각성(r = .37, *p* < .001), 지각된 유익성(r = .36, *p* < .001), 자기효능감(r = .59, *p* < .001)과 유의한 양의 상관관계로 나타났으며, 지각된 장애성과 유의한 음의 상관관계가 있는 것으로 나타났다(r = −.20, *p* = .017)(Table 4).

### 6. HPV 예방접종 의도에 영향을 미치는 요인

대상자의 예방접종 의도에 영향을 미치는 요인을 알아보기 위해 유의한 상관성이 확인된 변수인 일반적 특성 중 거주기간과 성 특성 중 첫 성경험 연령 그리고 건강신념 하부요인 중 지각된 민감성, 지각된 심각성, 지각된 유익성, 지각된 장애성, 자기효능감과 독립변수로 선정하고 예방접종 의도를 종속변수로 하여 모든 독립변수를 동시에 투입하는 입력(enter) 방법을 이용한 다중회귀분석을 실시하였다.

다중회귀분석 결과, 본 회귀모형은 통계적으로 유의하였고(F = 13.35, *p* < .001), HPV 예방접종 의도에 영향을 미치는 요인은 자기효능감(β = .48, *p* < .001), 거주기간 10년이상(β = .24, *p* = .003), 5년 이상~10년 미만(β = .20, *p* = .013), 지각된 장애성(β = −.19, *p* = .004), 지각된 민감성(β = .17, *p* = .028) 순으로 확인되었다. 해당 독립변수들은 예방접종 의도의 43.4%를 설명하는 것으로 나타났다(Table 5).

## 논의

본 연구에서는 국내에 거주하는 베트남 여성을 대상으로 건강신념모형을 통한 건강신념 정도를 파악하고, HPV 예방접종 의도에 영향을 미치는 요인을 확인하고자 수행하였다.

본 연구에서 HPV 예방접종 의도에 영향을 미치는 요인을 파악하기 위하여 회귀분석을 실시한 결과, 건강신념의 하부요인인 자기효능감이 HPV 예방접종 의도에 가장 큰 영향력을 미치는 변수로 나타났다. 행동방식의 변화에 유의한 영향을 주는 요인인 자기효능감을 증진하기 위한 전략은 간호학 등에서 많은 연구가 시행되고 있다. 결혼이주여성을 대상으로 한 자기효능감 관련 연구에서 높은 자기효능감을 가지고 있는 경우 진로 활동영역에서 직면하게 되는 여러 진로장벽의 요소에서 그것을 어떻게 해결할 것인지에 대한 전략적 사고를 찾고 실행하도록 한다고 밝혀졌으며[30], 자기효능감이 높을수록 건강 관련 삶의 질과 유방암 자가검진 수행이 높아짐을 확인했다[31,32]. 이는 자기효능감이 높을수록 HPV 예방접종 의도가 높음을 의미한다. 자궁경부암 예방행위 의도에 있어 자기효능감이 주요 예측 변인으로 나타난 선행 연구의 결과와 일치한 결과이다[27,29,33]. 또한, 대학생을 대상으로 신종감염병 예방행동의도를 연구한 Lee [34]와 기혼간호사의 자기효능감, 양육 스트레스 및 조직문화가 건강증진행위에 미치는 영향을 연구한 Jung과 Jung [35]의 연구와 건강 관련 삶의 질에 미치는 영향을 연구한 Chon 등[31]의 연구에서도 건강신념 하부요인 중 자기효능감이 건강증진행위에 가장 큰 영향을 미치는 요인으로 확인된 결과를 나타내어 본 연구 결과를 지지하였다.

본 연구에서 HPV 예방접종 의도에 두 번째로 큰 영향을 미친 요인은 한국 거주기간으로 한국 거주기간이 길수록 HPV 예방접종 의도가 높은 것으로 나타났다. 이는 외국인 유학생이 거주기간이 길어짐에 따라 아플 때 병원에 가는 것을 선택하는 비율이 높아진 Kwak [36]의 연구와 유사한 결과로 거주기간이 길어질수록 한국어 소통이 편해지고, 한국 문화에 적응하면서 의료서비스 이용과정에 두려움이 해소되어 이용 빈도가 증가로 이어졌을 것이다. 또한, 이러한 결과는 한국 거주 초기에는 건강관리에 무지하다가 건강에 대한 새로운 관점이 생기고, 점차 건강관리에 관심을 갖게 되는 일련의 한국 문화에 적응과정과 동일하며[37], 거주기간이 길수록 의료비 지출이 증가한다는 연구 결과[36]와도 같은 맥락이다. 결혼이주여성들은 점차 원문화를 상실하고 이주한 국가의 문화에 대한 적응 단계를 거치며, 한국인의 사회관계망에 편입될 가능성이 높아진다[38]. 따라서 국내 거주 베트남 여성들이 긍정적인 건강관리 신념을 형성할 수 있도록, 이주 초기부터 지역사회의 다문화센터와 이주여성 지원 단체를 활용한 지속적인 교육과 건강관리 인식 구축 프로그램을 제공하는 것이 필요하다.

HPV 예방접종 의도에 세 번째 영향요인은 지각된 장애성이었다. 베트남은 HPV 백신 무료접종이 시행되지 않고 있어, 백신 접종률이 12%로 낮은 편이다[39]. 또한, 결혼이주여성의 평균 초혼 나이는 29.5세로 내국인 여성보다 어리며, 남편과 연령차가 10세 이상인 부부의 비중이 38.1%로 높은 편이다[5]. 언어적 문제로 소통이 어려운 베트남 여성이 이주 초기에 의지할 수 있는 사람은 배우자인데, 남성 배우자가 아내의 전반적인 건강관리 지식 및 인식의 변화를 도모하는 것에는 한계가 있을 수 있다. 또한, 우리나라 결혼이주여성의 배우자를 대상으로 한 선행연구에서는 결혼이주여성이 HPV 백신 미접종의 주요 원인으로 HPV 백신에 대한 지식 부족과 비용문제를 보고하였던 바 있다[10,13]. 의학계에서는 중년남성으로의 접종대상 확대안을 권고하고 있으나[40] 아직 우리나라의 HPV 백신 국가 예방접종 대상에 남성은 포함되어있지 않다[3]. 따라서 우리나라에서 HPV에 대한 남성의 관심은 낮은 현실이다. 이러한 상황을 고려할 때, 기초적인 보건 지식을 습득할 수 있는 교육 과정을 마련하는 것이 근본적으로 필요하다. 이를 위해서는 지역사회 보건의료기관이나 외국인 종합지원센터가 중심이 되어, 사회적 측면에서 제도적 지원 방안을 마련해야 할 것이다.

마지막으로 지각된 민감성이 본 연구에서 HPV 예방접종 의도에 영향력을 미치는 요인으로 나타났다. 이는 지각된 민감성이 높을수록 예방접종 의도가 증가하는 것으로 확인된 많은 선행연구의 결과와 일치한다[20,33,41]. 다른 건강신념 하부요인에 비해 차순위 변수로 나타난 계기에는 HPV에 대한 위험성을 인식하고 있지만, 자신이 HPV에 감염될 것이라는 지각된 민감성이 낮게 인식되고 있는 경향이 있음을 알 수 있다. 베트남은 자궁경부암 스크리닝검사를 국가정책으로 아직 시행하고 있지 않은 나라이므로[6], 대상자들이 확인된 HPV 감염자를 인식하고 있을 가능성이 작다. 따라서 지각된 민감성을 높이기 위하여 자궁경부암이 국내 성인 여성에게 발생률 5위를 차지하는 흔한 질병으로 발생이 쉽다는 점을 강조하고, 건강하지 못한 성생활의 원인으로 인한 자궁경부암 발생 가능성의 관계를 인지하도록 할 필요가 있다.

본 연구의 제한점은 다음과 같다. 첫째, 충청북도 소재 국한된 지역에 거주하고 있는 베트남 여성을 대상으로 수행한 횡단적 연구로 일반화하는 데 제한점이 있다. 둘째, 본 연구 참여자의 대부분은 기혼이었으나, 다문화가정 내 배우자의 인식이나 역할 등은 조사하지 않아 이와 관련된 영향을 확인하지 못한 한계가 있다. 따라서 연구의 대상자를 확대하여 명확한 HPV 예방접종 관련 건강신념과 예방접종 의도의 인과관계를 규명할 수 있는 연구를 제언한다.

## 결론

본 연구는 국내 거주 베트남 여성의 HPV 예방접종 관련 건강신념이 예방접종 의도에 미치는 영향을 파악하기 위한 서술적 조사연구이다. 연구 결과 HPV 예방접종 의도에 영향력을 미치는 요인은 자기효능감, 거주기간, 지각된 장애성, 지각된 민감성으로 확인되었다. 본 연구는 증가추세에 있는 베트남 해외 이주여성을 대상으로 건강신념모형을 통한 건강신념 정도를 파악하고, HPV 예방접종 의도에 영향을 미치는 요인을 확인하여 향후 HPV 예방접종 의도 증진을 위한 기초자료를 제공하였다는 점에서 공중보건, 다문화 가정 건강지원 등 다양한 측면에서 의의가 있다.

향후 연구에서는 보다 다양한 지역의 국내 거주 베트남 여성을 대상으로 표본의 대표성을 확보할 수 있는 연구 설계를 통해 연구 결과의 일반화 가능성을 높일 필요가 있다. 또한, 예방접종 의도를 향상시키기 위한 구체적이고 실질적인 교육프로그램을 개발하고 적용하여 예방접종 의도의 향상을 검증하는 연구 수행이 필요하며, 나아가 본 연구에서 확인한 HPV 백신 접종에 영향을 미치는 요인 외에 HPV 백신 미접종에 대한 원인을 파악하여 근본적인 예방 접종률 향상을 이뤄낼 것을 제안한다. 마지막으로 HPV 백신 접종의 무료 대상 범위를 특정계층의 여성에 국한하지 않고 전연령 여성과 남성에게까지 확대하는 정책을 통해 예방접종에 대한 접근성을 높여 궁극적으로 청소년의 예방 접종률 상승에도 기여할 수 있기를 제언하는 바이다.

## Figures and Tables

**Table 1.** Differences in Vaccination Intentions According to General Characteristics, HPV, and Sexual Experience (N = 146)

Variables	Categories	n (%) or M ± SD		t/F	*p*
General characteristics					
Age (years)	< 30	53 (36.3)	4.40 ± 0.88	1.14	.323
	30~34	40 (27.4)	4.60 ± 0.67		
	≥ 35	53 (36.3)	4.58 ± 0.66		
		32.2 ± 6.08			
Educational status	High school	66 (45.2)	4.48 ± 0.81	0.53	.661
	Diploma	36 (24.7)	4.61 ± 0.65		
	Bachelor	34 (23.3)	4.44 ± 0.79		
	≥ Master	10 (6.8)	4.70 ± 0.68		
Region of origin	Metropolitan area	75 (51.4)	4.47 ± 0.72	−0.89	.377
	Rural area	71 (48.6)	4.58 ± 0.79		
Marital status	Married	106 (72.6)	4.56 ± 0.74	0.75	.475
	Single	29 (19.9)	4.48 ± 0.91		
	Others	11 (7.5)	4.27 ± 0.65		
Presence of children	Present	104 (71.2)	4.49 ± 0.76	−0.76	.449
	Absent	42 (28.8)	4.60 ± 0.73		
Residential period in Korea (years)	< 5^a^	35 (24.0)	4.23 ± 0.97	3.67	.028
	5~9^b^	61 (41.8)	4.64 ± 0.63		a < b‡
	≥ 10^c^	50 (34.2)	4.58 ± 0.67		
		8.13 ± 5.02			
Employment status	Employed	77 (52.7)	4.36 ± 0.91	−1.62	.109
	Unemployed	88 (47.3)	4.59 ± 0.67		
Income (won/month)	< 1 million	54 (37.0)	4.63 ± 0.65	1.00	.371
	1~ < 2 million	49 (33.6)	4.49 ± 0.68		
	≥ 2 million	43 (29.4)	4.42 ± 0.93		
Perceived economic status	Sufficient	22 (15.0)	4.55 ± 0.80	0.40	.673
	Average	101 (69.2)	4.54 ± 0.74		
	Insufficient	23 (15.8)	4.39 ±0.78		
HPV-related characteristics					
Awareness of HPV	Known	109 (74.7)	4.49 ± 0.78	0.35	.730
	Unknown	37 (25.3)	4.43 ± 0.88		
Experience of HPV vaccine	Yes	111 (76.0)	4.53 ± 0.73	1.49	.140
	No	35 (24.0)	4.27 ± 1.00		
Awareness of cervical cancer	Known	133 (91.1)	4.47 ± 0.80	−0.10	.917
	Unknown	13 (8.9)	4.50 ± 0.97		
Experience of cervical cancer screening	Yes	83 (56.8)	4.56 ± 0.68	1.40	.127
	No	63 (43.2)	4.33 ± 0.97		
Sexual experience-related characteristics					
Experience of coitus	Yes	135 (92.5)	4.50 ± 0.79	1.28	.203
	No	11 (7.5)	4.13 ± 0.99		
Age of first coitus experience^†^	< 20^a^	20 (14.9)	4.25 ± 1.07	3.65	.029
	20~24^b^	82 (61.2)	4.50 ± 0.74		a < c^‡^
	≥ 25^c^	32 (23.9)	4.78 ± 0.49		
		22.38 ± 2.88			
Number of sexual partners (people)^†^	1	82 (61.2)	4.54 ± 0.67	0.02	.976
	2	36 (26.9)	4.53 ± 0.81		
	≥ 3	16 (11.9)	4.50 ± 0.82		
Frequency of contraceptive use^†^	All the time	41 (30.6)	4.56 ± 0.67	0.63	.599
	Usually	23 (17.2)	4.70 ± 0.47		
	Sometimes	42 (31.3)	4.48 ± 0.77		
	Never	28 (20.9)	4.45 ± 0.87		

HPV = Human papillomavirus; M = Mean; SD = Standard deviation. ^†^Except for non-response among those with sexual experience (N = 134); ^‡^Scheffé’s test.

**Table 2.** Health Beliefs Subconstructs and HPV Vaccination Intentions (N = 146)

Variables	Item	M ± SD	Min~Max	Range
Perceived susceptibility	5	3.82 ± 0.90	1.60~5.00	1~5
Perceived severity	4	4.14 ± 0.84	1.75~5.00	1~5
Perceived benefits	5	4.06 ± 0.77	2.00~5.00	1~5
Perceived barriers	6	2.90 ± 0.80	1.00~5.00	1~5
Self-efficacy	3	4.29 ± 0.74	2.67~5.00	1~5
Vaccination intentions	1	4.52 ± 0.75	2.00~5.00	1~5

HPV = Human papillomavirus; M = Mean; SD = Standard deviation.

**Table 3.** Differences of Health Beliefs Subconstructs According to General Characteristics, HPV, and Sexual Experience (N = 146)

Variables	Categories	n (%)	Perceived susceptibility		Perceived severity		Perceived benefits		Perceived barriers		Self-efficacy	
			M ± SD	t/ F(*p*)	M ± SD	t/F (*p*)	M ± SD	t/F (*p*)	M ± SD	t/F (*p*)	M ± SD	t/F (*p*)
General characteristics												
Age (years)	< 30	53 (36.3)	3.66 ± 0.98	1.54 (.218)	4.02 ± 0.86	0.73 (.484)	3.89 ± 0.75	2.50 (.085)	2.88 ± 0.67	1.28 (.282)	4.25 ± 0.82	0.19 (.826)
	30~34	40 (27.4)	3.87 ± 0.85		4.19 ± 0.80		4.09 ± 0.75		2.76 ± 0.89		4.34 ± 0.66	
	≥ 35	53 (36.3)	3.95 ± 0.84		4.20 ± 0.86		4.22 ± 0.77		3.02 ± 0.84		4.28 ± 0.72	
Educational status	High school	66 (45.2)	3.87 ± 0.89	0.95 (.963)	4.24 ± 0.79	1.27 (.289)	4.19 ± 0.75	1.53 (.209)	2.96 ± 0.83	2.62 (.053)	4.30 ± 0.77	0.41 (.744)
	Diploma	36 (24.7)	3.79 ± 0.87		3.98 ± 0.87		4.02 ± 0.74		2.96 ± 0.68		4.28 ± 0.75	
	Bachelor	34 (23.3)	3.79 ± 0.99		4.18 ± 0.77		3.94 ± 0.75		2.90 ± 0.86		4.21 ± 0.74	
	≥ Master	10 (6.8)	3.76 ± 0.90		3.83 ± 1.26		3.76 ± 1.01		2.23 ± 0.50		4.50 ± 0.59	
Region of origin	Metropolitan area	75 (51.4)	3.85 ± 0.93	0.34 (.734)	4.08 ± 0.87	−0.86 (.389)	4.08 ±0.79	.32 (.751)	2.90 ± 0.76	0.01 (.994)	4.17 ± 0.76	−1.97 (.050)
	Rural area	71(48.6)	3.80 ± 0.87		4.20 ± 0.81		4.0 4 ± 0.75		2.90 ± 0.84		4.41 ± 0.70	
Marital status	Married	106 (72.6)	3.85 ± 0.92	0.91 (.407)	4.13 ± 0.85	0.01 (.995)	4.10 ± 0.76	0.97 (.383)	2.92 ± 0.84	0.31 (.737)	4.34 ± 0.71	1.24 (.294)
	Single	29 (19.9)	3.86 ± 0.84		4.13 ± 0.84		3.88 ± 0.76		2.79 ± 0.71		4.21 ± 0.88	
	Others	11 (7.5)	3.47 ± 0.90		4.16 ± 0.88		4.11 ± 0.85		2.92 ± 0.65		4.00 ± 0.62	
Presence of children	Present	104 (71.2)	3.81 ± 0.92	−0.37 (.356)	4.11 ± 0.86	−0.61 (.543)	4.09 ± 0.78	0.65 (.517)	2.89 ± 0.83	−0.26 (.793)	4.26 ± 0.73	−0.58 (.564)
	Absent	42 (28.8)	3.87 ± 0.85		4.20 ± 0.81		4.00 ± 0.73		2.92 ± 0.72		4.34 ± 0.78	
Residential period in Korea (years)	< 5	35 (24.0)	3.86 ± 0.96	0.25 (.777)	4.11 ± 0.84	0.04 (.966)	4.10 ± 0.73	0.32 (.727)	2.94 ± 0.86	0.31 (.737)	4.24 ± 0.85	0.19 (.831)
	5~9	61 (41.8)	3.76 ± 0.85		4.13 ± 0.83		4.00 ± 0.73		2.84 ± 0.73		4.33 ± 0.72	
	≥ 10	50 (34.2)	3.87 ± 0.93		4.16 ± 0.88		4.10 ± 0.76		2.94 ± 0.83		4.27 ± 0.69	
Employment status	Employed	77 (52.7)	3.80 ± 0.88	−0.29 (.770)	4.07 ± 0.82	−0.92 (.360)	4.07 ± 0.73	0.16 (.871)	2.83 ± 0.77	−1.09 (.276)	4.32 ± 0.76	0.60 (.549)
	Unemployed	88 (47.3)	3.85 ± 0.93		4.20 ± 0.87		4.05 ± 0.81		2.97 ± 0.82		4.25 ± 0.73	
Income (won/month)	< 1 million	54 (37.0)	3.90 ± 0.93	2.64 (.074)	4.20 ± 0.83	0.36 (.698)	4.14 ± 0.77	0.64 (.528)	2.85 ± 0.82	0.16 (.854)	4.35 ± 0.70	0.42 (.658)
	1~ < 2 million	49 (33.6)	3.59 ± 0.87		4.14 ± 0.68		4.05 ± 0.71		2.92 ± 0.78		4.22 ± 0.78	
	≥ 2 million	43 (29.4)	4.00 ± 0.88		4.05 ± 1.01		3.97 ± 0.83		2.93 ± 0.80		4.28 ± 0.75	
Perceived economic status	Sufficient	22 (15.0)	4.04 ± 0.87	1.09 (.337)	4.18 ± 0.99	1.68 (.189)	4.20 ± 0.81	2.44 (.091)	2.80 ± 1.00	0.23 (.792)	4.44 ± 0.73	0.61 (.544)
	Average	101 (69.2)	3.75 ± 0.90		4.06 ± 0.83		3.97 ± 0.78		2.91 ± 0.72		4.25 ± 0.77	
	Insufficient	23 (15.8)	3.93 ± 0.93		4.41 ± 0.68		4.32 ± 0.63		2.95 ± 0.94		4.30 ± 0.64	
HPV related characteristics												
Awareness of HPV	Yes	109 (74.7)	3.88 ± 0.87	1.24 (.216)	4.18 ± 0.79	0.91 (.370)	4.08 ± 0.70	0.43 (.670)	2.83 ± 0.75	−1.88 (.063)	4.32 ± 0.69	0.89 (.378)
	No	37 (25.3)	3.66 ± 0.98		4.01 ± 1.00		4.01 ± 0.96		3.11 ± 0.91		4.18 ± 0.88	
Experience of HPV vaccine	Yes	111 (76.0)	3.95 ± 0.85	3.02 (.003)	4.19 ± 0.78	1.22 (.230)	4.08 ± 0.72	0.60 (.550)	2.83 ± 0.78	−1.82 (.071)	4.35 ± 0.70	1.66 (.104)
	No	35 (24.0)	3.43 ± 0.96		3.96 ± 1.00		3.98 ± 0.92		3.11 ± 0.82		4.09 ± 0.85	
Awareness of cervical cancer	Yes	133 (91.1)	3.88 ± 0.86	2.40 (.018)	4.14 ± 0.85	0.43 (.666)	4.05 ± 0.77	−0.76 (.447)	2.86 ± 0.78	−2.03 (.045)	4.31 ± 0.74	1.20 (.234)
	No	13 (8.9)	3.26 ± 1.10		4.04 ± 0.83		4.22 ± 0.81		3.32 ± 0.91		4.05 ± 0.74	
Experience of cervical cancer screening	Yes	83 (56.8)	4.00 ± 0.84	2.71 (.008)	4.32 ± 0.74	3.12 (.002)	4.19 ± 0.70	2.34 (.021)	2.98 ± 0.88	1.44 (.151)	4.38 ± 0.69	1.74 (.085)
	No	63 (43.2)	3.60 ± 0.94		3.89 ± 0.91		3.89 ± 0.83		2.79 ± 0.67		2.79 ± 0.67	
Sexual experience related characteristics												
Experience of coitus	Yes	135 (92.5)	3.82 ± 0.91	−0.05 (.960)	4.14 ± 0.85	0.37 (.714)	4.09 ± 0.77	1.92 (.057)	2.90 ± 0.80	0.54 (.592)	4.30 ± 0.71	0.77 (.459)
	No	11 (7.5)	3.84 ± 0.75		4.05 ± 0.74		3.64 ± 0.64		2.77 ± 0.73		4.06 ± 1.03	
Age of first coitus experience^†^	< 20	20 (14.9)	3.73 ± 0.98	0.86 (.426)	3.93 ± 0.89	1.12 (.330)	3.79 ± 0.86	2.05 (.132)	2.74 ± 0.56	0.52 (.596)	3.97 ± 0.88	3.31 (.040)
	20~24	82 (61.2)	3.77 ± 0.88		4.14 ± 0.83		4.12 ± 0.72		2.95 ± 0.85		4.32 ± 0.69	
	≥ 25	32 (23.9)	4.00 ± 0.95		4.29 ± 0.89		4.22 ± 0.83		2.89 ± 0.81		4.48 ± 0.62	
Number of sexual partners (people)^†^	1	82 (61.2)	3.79 ± 0.98	0.33 (.719)	4.17 ± 0.81	0.21 (.807)	4.09 ± 0.77	0.05 (.954)	3.00 ± 0.84	1.37 (.257)	4.27 ± 0.71	0.56 (.574)
	2	36 (26.9)	3.93 ± 0.80		4.06 ± 1.00		4.08 ± 0.77		2.74 ± 0.64		4.30 ± 0.74	
	≥ 3	16 (11.9)	3.76 ± 0.78		4.17 ± 0.73		4.15 ± 0.79		2.82 ± 0.92		4.48 ± 0.70	
Frequency of contraceptive use^†^	All the time	41 (30.6)	3.86 ± 1.00	1.28 (.283)	4.20 ± 0.86	0.31 (.819)	4.14 ± 0.74	0.18 (.912)	3.00 ± 0.90	2.53 (.060)	4.40 ± 0.67	0.67 (.571)
	Usually	23 (17.2)	3.50 ± 0.93		4.12 ± 0.91		4.02 ± 0.81		2.59 ± 0.47		4.39 ± 0.64	
	Sometime	42 (31.3)	3.87 ± 0.90		4.18 ± 0.73		4.13 ± 0.75		2.83 ± 0.83		4.22 ± 0.76	
	Never	28 (20.9)	3.97 ± 0.77		4.02 ± 0.99		4.05 ± 0.84		3.16 ± 0.76		4.22 ± 0.77	

HPV = Human papillomavirus; M = Mean; SD = Standard deviation. ^†^Except for non-response among those with sexual experience (N = 134).

**Table 4.** Correlation between HPV Vaccination Related Health Beliefs Subconstructs and Self Vaccination Intentions (N = 146)

Variables	1	2	3	4	5
	r (*p*)				
1. Perceived susceptibility	1	-	-	-	-
2. Perceived severity	.56 (< .001)	1	-	-	-
3. Perceived benefits	.46 (< .001)	.69 (< .001)	1	-	-
4. Perceived barriers	.16 (.058)	.05 (.553)	.12 (.142)	1	-
5. Self-efficacy	.33 (< .001)	.41 (< .001)	.51 (< .001)	−.08 (.335)	1
6. Vaccination intentions	.35 (< .001)	.37 (< .001)	.36 (< .001)	−.20 (.017)	.59 (< .001)

HPV = Human papillomavirus.

**Table 5.** Factors Affecting HPV Vaccination Intentions (N = 146)

Variables	B	SE	β	t	*p*
(constant)	1.85	0.37		5.01	< .001
Perceived susceptibility	0.15	0.07	.17	2.23	.028
Perceived severity	0.08	0.08	.09	0.94	.347
Perceived benefits	−0.03	0.09	−.03	−0.26	.794
Perceived barriers	−0.18	0.06	−.19	−2.92	.004
Self-efficacy	0.49	0.08	.48	6.33	< .001
Residential period in Korea (years) (5 ≤, < 10)	0.03	0.12	.20	2.51	.013
Residential period in Korea (years) (≥ 10)	0.41	0.13	.24	3.04	.003
Age of first coitus experience^†^ (20 ≤, < 25)	0.02	0.13	.01	0.17	.866
Age of first coitus experience^†^ (≧ 25)	0.19	0.15	.11	1.13	.198
R² = .47					
Adjusted R² (%) = 43.4					
F (*p*) = 13.35 (< .001)					

HPV = Human papillomavirus; SE = Standard error. ^†^Except for non-response among those with sexual experience (N = 134).

## Data Availability

The data supporting this study's findings are accessible from the corresponding author upon reasonable request. Due to privacy and ethical considerations, the data are not publicly available.
